# Administration of a Recombinant Fusion Protein of IFN-γ and CD154 Inhibited the Infection of Chicks with *Salmonella enterica*

**DOI:** 10.3390/vetsci12020112

**Published:** 2025-02-02

**Authors:** Jingya Zhang, Guofan Ren, Wei Li, Honglin Xie, Zengqi Yang, Juan Wang, Yefei Zhou, Xinglong Wang

**Affiliations:** 1 College of Veterinary Medicine, Northwest A&F University, Yangling 712100, China; zhangjingya_@nwafu.edu.cn (J.Z.); renguofan@nwafu.edu.cn (G.R.); 1546280879@nwafu.edu.cn (W.L.); honglinxie@nwafu.edu.cn (H.X.); yzq1106@nwsuaf.edu.cn (Z.Y.); juan.wang@nwafu.edu.cn (J.W.); 2 Department of Life Science, Nanjing Xiaozhuang University, Nanjing 211171, China

**Keywords:** *Salmonella* Typhimurium, IFN-γ, CD154, cecum, inflammation

## Abstract

The cytokines IFN-γ and CD154 have been well established, and they play pivotal roles in immune protection against *Salmonella* in mice, but their effects and specific mechanisms in *Salmonella*-infected chickens are less understood. In this study, we conducted animal experiments to screen the highly immunoprotective chIFN-γ-chCD154 fusion protein compared with single protein chIFN-γ or chCD154 in white Leghorn chickens. The results showed that compared with separate pretreatments with chIFN-γ and chCD154, the fusion protein, chIFN-γ-chCD154, synergistically increased survival of infected chickens, reduced bacterial load in feces and organs, and attenuated pathological damage to the liver and cecum. Pretreatment with chIFN-γ-chCD154 also increased humoral immune responses, expression of the tight junction proteins zo-1, occludin, and claudin-1, and the relative abundance of *Enterococcus_cecorum*, *Lactobacillus_helveticus*, and *Lactobacillus_agilis*, which protect against intestinal inflammation. Compared with single protein pretreatment, chIFN-γ-chCD154 significantly upregulated STAT1, IRF1, and GBP1 in infected chickens while decreasing mRNA expression of TLR4, MyD88, NF-κB, TNF-α, IL-6, and IL-1β. In summary, damage to the cecal epithelial barrier and the inflammation induced by *S. typhimurium* infection was alleviated by chIFN-γ-chCD154 pretreatment through a mechanism involving the TLR4/MyD88/NF-κB and IFN-γ/STAT/IRF1/GBP1 pathways.

## Simple Summary

*Salmonella* infections pose a significant risk to poultry health, affecting both animal welfare and food safety. While the immune functions of two proteins, IFN-γ and CD154, are well known in mice, their roles in chickens have not been thoroughly explored. This study focuses on a fusion protein combining chicken IFN-γ (chIFN-γ) and CD154 (chCD154) and its protective effects in chickens infected with *Salmonella*. Our results demonstrate that the fusion protein significantly improved survival rates, reduced bacterial loads, and lessened tissue damage compared with the use of either protein alone. Additionally, it enhanced immune responses and strengthened the gut barrier, which is crucial for protecting against infection. This research highlights the potential of chIFN-γ-chCD154 as a promising approach to boost immunity and control *Salmonella* infection in poultry.

## 1. Introduction

*Salmonella*, a Gram-negative enteric pathogen in the *Enterobacteriaceae* family, causes over one million infections in humans and livestock per year globally and poses extensive challenges to healthcare and economics [1]. Poultry is considered to be the primary reservoir of *Salmonella* infections [2], and food prepared from *Salmonella-*contaminated poultry is a major source of salmonellosis, which is a serious threat to human health [2,3]. To date, *Salmonella* comprises over 3000 serotypes, with *Salmonella enterica* serovar Typhimurium, (*S.* Typhimurium) being the dominant serotype associated with salmonellosis worldwide [4]. Although various preventive strategies have been used to inhibit *S.* Typhimurium infection in chickens, such as vaccination programs and microflora-modulating feed additives in the poultry breeding industry, the hunt for more effective treatments is still ongoing. In this study, we have focused on two very important proteins in the antibacterial immune defense system, type II interferon gamma (IFN-γ) and CD40 ligand (CD154).

IFN-γ and CD154 have been characterized as critical mediators in the immune response that help the host defend against viral and bacterial infections [5,6,7]. Exogenous IFN-γ induces a host immune response and has an essential role in preventing and eliminating the invasion of *Salmonella* [8]. CD154 (CD40 ligand), a member of the TNF superfamily, regulates the host humoral and cellular immune responses by binding to the CD40 receptor [7]. *Salmonella*-infected macrophages treated with exogenous CD154 significantly upregulated IL-12p70 mRNA expression, promoted the production of IFN-γ, and enhanced macrophage phagocytosis of pathogens [9]. After injecting a lethal dose of *Salmonella* into mice, exogenous CD154 treatment significantly prolonged their survival time [10]. IFN-γ combined with CD154 improved host resistance to *Mycobacterium tuberculosis* infection [11]. However, the application of chIFN-γ-CD154 to prevent *Salmonella* infection in chickens has rarely been reported. In this study, pretreatment of chickens with the fusion protein, chIFN-γ-chCD154, inhibited *S.* Typhimurium infection and thus could serve as a novel therapeutic for preventing pathogenic bacterial infection in chickens.

The host intestine contains a complex microbial community with a decisive role in maintaining the normal physiology of the host, including the formation of intestinal structure and morphology, development of the immune system, digestion and utilization of nutrients, and resistance to pathogens [12,13,14]. The intestinal microbiota influences the homeostasis of the host immune system by regulating the innate myeloid and lymphoid cells and promoting intestinal immune homeostasis [15]. A previous study reported that *S.* Typhimurium infection changed the structure of the intestinal microbiota, resulting in immunological dysregulation and intestinal inflammation [16]. Normal intestinal microbiota can decrease the risk of pathogen infection by direct competitive microbial antagonism and by inducing the host’s immune responses [17]. However, there has been no scientific evidence about how chIFN-γ-CD154 affects host health by altering the intestinal microbiota. Thus, 16S rRNA gene sequencing and transcriptomic sequencing were conducted to investigate the effects of chIFN-γ-CD154 on the structure of the microbial community in the gut in white Leghorn chickens. Additionally, pretreatment with a chIFN-γ-CD154 fusion protein was also evaluated to determine whether it could prevent the cecum microbiota structure from being damaged by *S.* Typhimurium infection.

It has been widely demonstrated that tight junction (TJ) proteins play an indispensable role in pathogen infection, because disruption of TJs markedly increases paracellular permeability and polarity defects. This facilitates viral and bacterial entry through damaged TJs and causes inflammation of host intestinal mucosa, further impairing the integrity of the intestinal epithelial barrier and increasing bacterial spread [18,19]. Toll-like receptor 4 (TLR4) is a transmembrane signaling receptor that links innate immunity to adaptive immunity [20]. It belongs to the TLR family that recognizes and is activated by bacterial lipopolysaccharide (LPS), which is the main component of the cell walls of Gram-negative bacteria like *Salmonella* [20]. TLR4 recognition of LPS stimulates MyD88-dependent nuclear factor kappa B (NF-κB) activation, which induces the overproduction of pro-inflammatory cytokines, resulting in tissue damage, organ dysfunction, and even host death [21]. TLR-induced inflammation is mainly signaled through the TLR4-activated myeloid differentiation primary response protein 88 (MyD88)-dependent NF-κB (TLR4/MyD88/NF-κB) pathway [22,23]. The inhibition of TLR4 reduces the expression of MyD88, suppresses activation of the NF-κB signaling pathway, reduces the secretion of pro-inflammatory cytokines, and alleviates tissue damage [23,24]. A previous study suggested that IFN-γ signaling activated STAT1-IRF1–mediated induction of guanylate binding proteins (GBPs), which resulted in cell death that prevented bacterial spread in vivo [25]. Thus, the mechanism whereby chIFN-γ-chCD154 was able to control *S.* Typhimurium infection was preliminarily investigated in this study.

## 2. Materials and Methods

### 2.1. Expression Vectors, Bacteria, and Animals

The pCold vector was purchased from HaiGene Biotech Co., Ltd. (Haerbin, China). *Salmonella enterica* serovar Typhimurium, ATCC14028, was obtained from Shanghai Xinyu Biotechnology Co., Ltd. (Shanghai, China) and cultured in Luria–Bertani (LB) broth at 37 °C and 220 rpm. One hundred and fifty 1-day-old specific pathogen-free (SPF) white Leghorn layer chickens were obtained from Jinan Spafas Poultry Co., Ltd. (Jinan, China), and raised in isolation facilities. Water and food were provided ad libitum, and no antibiotics were administered to animals in this study.

### 2.2. Expression of Recombinant Proteins

Total RNA was extracted from chicken spleens with TRIzol regent (Solarbio, Beijing, China), and cDNA was prepared with a first-strand synthesis kit (StarScript II-RT, GeneStar, Beijing, China). For the chicken IFN-γ sequence (GenBank No. FJ788637.1) and the chicken CD154 sequence (GenBank No. AJ243435), the two fragments were amplified by the relevant forward and reverse primers and then cloned into the Not I- and BamH I-digested expression vector pCold using a seamless cloning technique to generate pCold-chIFN-γ and pCold-chCD154. The chCD154 was inserted into the Not I- and BamH I-digested pCold-chIFN-γ vector by a seamless cloning technique to generate the recombinant plasmid, pCold-chIFN-γ-chCD154. The primers used are listed in Table 1.

The PCR products were confirmed by sequencing.

The above three recombinant plasmids (pCold-chIFN-γ, pCold-chCD154, and pCold-chIFN-γ-chCD154) were transduced into *E. coli* BL21 (DE3), and protein expression was turned on by the addition of isopropyl-β-D-1-thiogalactopyranoside (Sigma, MO, USA) to a final concentration of 0.5 mM and incubation at 16 °C for 18 h. A nickel-IDA affinity chromatography column (Beytotime, Shanghai, China) was used to purify the His-tagged recombinant proteins from the soluble fraction of the bacterial lysates. SDS-PAGE and Western blotting were performed to confirm the expression of the recombinant proteins. Mouse anti-6×His monoclonal antibody (Beytotime, Shanghai, China) was used as the primary antibody, and after thorough washing, the PVDF membranes were incubated with ECL reagents (Thermo Scientific, Wilmington, IL, USA) and the bands were imaged with a ChemiDoc TMTouch Imaging System (Bio-Rad, Hercules, CA, USA). The purified proteins were freeze-dried (Xinzhi, Zhejiang, China) and diluted in ddH_2_O for use.

### 2.3. Animal Experiments

**Figure 1:**
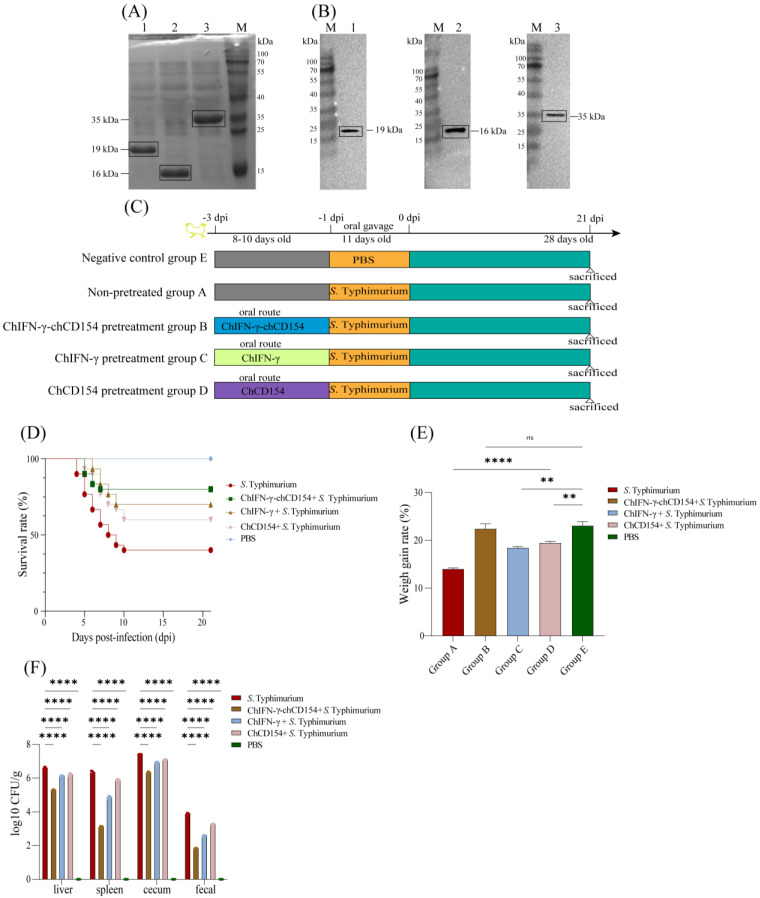
ChIFN-γ-chCD154 pretreatment alleviated *S.* Typhimurium infection in chickens. (**A**) SDS-PAGE and (**B**) Western blot analysis of chIFN-γ, chCD154, and chIFN-γ-chCD154. Lane M: protein molecular weight standard (The original images are listed in the Supplementary Materials); Lane 1: 19 kDa chIFN-γ protein; Lane 2: 16 kDa chIFN-γ-chCD154 protein; Lane 3: 35 kDa chCD154 protein. (**C**) Schematic illustration of animal experiments. (**D**) Survival rates. (**E**) The weight gain rates (%). The data are expressed as the mean ± SD (n = 3 per group). ** *p* < 0.01, **** *p* < 0.0001, and ns = not significant applies to the *S.* Typhimurium-infected groups pretreated with chIFN-γ, chCD154, or chIFN-γ-chCD154 compared with the control group. (**F**) Bacterial load in liver, spleen, cecum, and feces of chickens infected with *S.* Typhimurium. The data are the mean ± SD (n = 3 per group). **** *p* < 0.0001 are the groups of *S.* Typhimurium pretreated by chIFN-γ, chCD154, or chIFN-γ-chCD154 versus the infected control group pretreated only with PBS.

The 150 one-day-old SPF white Leghorn chicks were randomly allocated into five groups of thirty chickens each (Figure 1C). The experiments were conducted from the 1st to the 24th days of age of the chicks. The one-day-old chicks in groups B, C, and D were pretreated with 0.2 mL of PBS containing 40 μg of recombinant protein via the oral route, once a day for three days before *S.* Typhimurium infection. Each four-day-old chick in the *S.* Typhimurium-infected groups (A, B, C, D) was administered with 200 μL of PBS containing 3.76 × 10^8^ CFU/mL of *S.* Typhimurium by gavage. Also, the negative control (Group E) four-day-old chicks were given 200 μL of PBS alone by gavage. Group A (non-pretreated group) received *S.* Typhimurium inoculum only. Group B (chIFN-γ-chCD154 pretreatment group) received 40 μg/chick of chIFN-γ-chCD154 + *S.* Typhimurium. Group C (chIFN-γ pretreatment group) received 40 μg/chick of chIFN-γ + *S.* Typhimurium. Group D (chCD154 pretreatment group) received 40 μg/chick of chCD154 + *S.* Typhimurium. Group E (negative control, uninfected, non-pretreated group) received 200 μL of PBS alone.

The chickens’ survival rate was monitored daily. The chickens were weighed at the beginning of the experiments and at the end of the experiments to calculate the percent weight gain.

### 2.4. Collection of Samples

At 21 days post-infection (dpi), all surviving chickens were euthanized, and the liver, spleen, cecum and cecal contents were aseptically removed from each group using sterile tools and placed in sterile sample bags for subsequent experiments. In addition, fecal samples from each group were collected. All of the samples collected were stored at −80°C for future analysis. The liver, spleen, cecum and fecal samples from three chickens of each group were subjected to determination of *S.* Typhimurium numbers. The ceca of three chickens from each group was carefully cut open longitudinally and gently flushed with ice-cold PBS to obtain mucosal samples for the measurement of secretory IgA (sIgA). Total RNA was extracted from cecal tissues of three chickens from each group. The liver and cecal tissue samples were fixed in 4% paraformaldehyde for histological examination. The total proteins of the cecum were extracted with RIPA lysis buffer (Solarbio, Beijing, China) containing 1% PMSF (Beyotime, Shanghai, China) and 1% phosphatase inhibitor cocktail (Beyotime, Shanghai, China) for Western blotting. The cecal contents from three chickens were snap-frozen in liquid nitrogen and stored for later 16S rRNA gene sequencing. The cecal tissues from six chickens were snap-frozen in liquid nitrogen and stored for transcriptomics profiling. Blood samples were drawn from the wing veins of three chickens in each group using serum tubes without anticoagulants. To obtain serum, the blood samples were allowed to clot and then were centrifuged at 2500× *g* for 5 min and stored at −20 °C.

### 2.5. Enzyme-Linked Immunosorbent Assay (ELISA)

An ELISA assay was used to assess the anti-*S.* Typhimurium IgG levels in the chickens as previously described, with slight modifications [26]. Briefly, *S.* Typhimurium (10^8^ CFU/mL) was washed and lysed for 5 min at 4 °C. The lysates were centrifuged at 8000× *g* for 5 min, and the supernatants were collected and stored at −80°C. The 96-well ELISA microplates (Corning, NY, USA) were coated with 100 μL/well of a 20 μg/mL solution of the antigen in coating buffer (0.015 M Na_2_CO_3_, 0.035 M NaHCO_3_) for the determination of anti-*S.* Typhimurium antibodies. Serum samples were 100-fold diluted in PBST with 1% BSA in triplicate (100 µL/well). Absorbance values were read at 450 nm with an ELISA plate reader (Bio Tek EL311sx Autoreader, Bio Tek, Winooski, VT, USA).

### 2.6. Measurement of Secretory IgA (sIgA)

The cecum was carefully cut open longitudinally and gently flushed with ice-cold PBS. Mucosal samples were scraped off with a sterile glass microscope slide and added to tubes containing PBS and 0.2 M EDTA (pH = 7.3). The supernatants were obtained after centrifugation at 8000× *g* for 10 min at 4 °C. The sIgA levels were measured with a commercial ELISA kit (Fankew, Shanghai, China).

### 2.7. Determination of S. Typhimurium Numbers in Feces and Internal Organs

At 21 dpi, liver, spleen, cecum, and fecal samples were obtained from three chickens randomly selected from each group. The 0.1g samples were homogenized in 1 mL of sterile PBS for 60 s using an IKA T10 basic Ultra-Turrax^®^ homogenizer (IKA, Königswinter, Germany). The homogenate was then centrifuged at 10,000× *g* for 30 min at 4 °C (5804/5804-R centrifuge, Eppendorf, Enfield, CT, USA). Supernatants were diluted with serial 10-fold dilutions, and 100 µL aliquots were plated on *Salmonella Shigella* (SS) agar plates and incubated at 37 °C for 24 h to determine *S.* Typhimurium colony forming units (CFUs), which were presence with colorless colonies (1–3 mm) with a black center [27]. Three replicates were averaged and expressed as the mean ± standard error of the mean log_10_ CFU/g fecal matter or tissue.

### 2.8. RNA Extraction and Determination of Relative RNA Expression by Real-Time Quantitative Polymerase Chain Reaction (RT-qPCR)

Total RNA was isolated from the cecum with TRIzol reagent (Solarbio, Beijing, China), and concentration was measured with a Nanodrop spectrophotometer (Thermo Scientific, Wilmington, DE, USA). The StarScript II-RT kit (GeneStar, Beijing, China) was used to generate cDNA. RT-qPCR was performed in triplicate with 2× RealStar Fast SYBR qPCR Mix (GeneStar, Beijing, China) in a TL-988 real-time PCR system (Tianlong, Xi’an, China). The 20 μL reaction contained 10 μL of 2× RealStar Fast SYBR qPCR mix, 0.5 μL of each primer, cDNA (100 ng), and RNase-free water. The thermocycling conditions for amplification were: 50 °C for 2 min; 95 °C for 10 min; and 40 cycles of 95 °C for 15 s and 60 °C for 60 s. CT values were normalized to the averaged readings for *β-actin* and determined by the 2^−ΔΔCT^ method. The primers used for amplifying *IL-1β*, *IL-6*, *IL-12*, *TNF-α*, *zo-1*, *occludin*, *claudin-1*, *STAT1*, *IRF1*, and *GBP1* are listed in Table 2.

### 2.9. Western Blot Protein Assay

Total protein was purified from cecal samples with RIPA lysis buffer (Solarbio, Beijing, China) containing 1% PMSF (Beyotime, Shanghai, China) and 1% phosphatase inhibitor cocktail (Beyotime, Shanghai, China). The protein concentrations were determined by a BCA protein assay kit (Beyotime, Shanghai, China). Equal amounts of proteins were separated by SDS-PAGE processing and electrotransferred to PVDF membranes. After blocking, the membranes were incubated with the following primary antibodies overnight at 4 °C: zo-1 (1:1000, Santa Cruz Biotechnology, Santa Cruz, CA, USA), occludin (1:1000, Abcam, Cambridge, MA, UK), claudin (1:1000, Sigma, St. Louis, MO, USA), STAT1 (1:1000, Proteintech Group, Wuhan, China), IRF1 (1:1000, LifeSpan BioSciences, Seattle, MA, USA), GBP1 (1:500, stored by our lab), and β-actin (1:2000, Proteintech Group, Wuhan, China), incubated at 4 °C for 24 h. After washing with TBST, the membranes were incubated with HRP-conjugated anti-mouse IgG secondary antibody (1:2000, Proteintech Group, Wuhan, China) or HRP-conjugated anti-rabbit IgG secondary antibody (1:2000, Proteintech Group, Wuhan, China) for 1 h at 37 °C. The target protein bands were visualized with an ECL kit (Beyotime, Shanghai, China), and image intensity was quantitated with an AlphaImager 2200 (Alpha Innotech, San Leandro, CA, USA).

### 2.10. Histopathological Examination

At 21 dpi, liver and cecal tissues of chickens from the non-pretreated group, the negative control group, and the chIFN-γ, chCD154, and chIFN-γ-chCD154 pretreatment groups were collected and fixed in 4% paraformaldehyde for 24 h at 4 °C. Samples were dehydrated, paraffin-embedded, cut into 4 μm sections, and stained with hematoxylin and eosin (Yike, Xi’an, China). The stained tissue sections were evaluated for histological pathology by microscopic examination at 400× (Olympus IX70, Olympus, Tokyo, Japan).

### 2.11. 16S rRNA Gene Sequencing Analyses

For 16S rRNA sequencing, at 21 dpi, cecal contents were collected from three randomly selected chickens of the non-pretreated group, the chIFN-γ-chCD154 pretreatment group, and the negative control group (UNPT, T, and NC groups), then stored at −80°C. The genomic DNA was isolated from the samples with a QIAmp stool DNA mini kit (Qiagen, Hilden, Germany). The V3–V4 16S rRNA regions were amplified with the following primers:

Forward: 5′-GTGCCAGCMGCCGCGGTAA-3′

Reverse: 5′-CCGTCAATTCCTTTGAGTTT-3′

on an ABI GeneAmp^®^ 9700 PCR thermocycler system (ABI, Foster City, CA, USA). The sequencing library was then prepared using a TruSeq NanoDNA LT library prep kit (Illumina, San Diego, CA, USA), and the library was sequenced and analyzed on a NovaSeq 6000 Illumina platform (Shanghai Personal Biotechnology Co., Ltd., Shanghai, China) to obtain the raw data.

The online MG-RAST service was used to assemble and annotate the raw data. Application of the DADA2 method for quality filtering, de-noising, splicing, and de-chimerizing yielded high-quality sequence reads that were clustered into operational taxonomic units (OTUs) with 97% similarity. The Greengenes database was used to annotate the OTUs. The FASTQ sample files were submitted to the NCBI (ID: PRJNA944214). The SILVA database was selected for the taxonomic assignment of OTUs. The Shannon, Chao1, observed species, and Simpson indices were used to determine the alpha diversity of the species in the samples. The non-metric multidimensional scaling (NMDS) analysis was performed on the Bray–Curtis distance matrix using an R script, and the compositional differences of microbial communities were displayed through a two-dimensional sequencing diagram. The R studio program (version 4.0.2) was used to perform statistical analyses and visualizations.

### 2.12. Cecal Tissue Transcriptome Profiling

At 21 dpi, six chickens from each group were euthanized for the collection of cecal tissue. Total RNA was extracted from cecal tissues with TRIzol regent (Solarbio, Beijing, China); the concentration and quality were measured with a NanoDrop spectrophotometer (Thermo Scientific, Wilmington, DE, USA). A NovaSeq 6000 platform (Illumina, San Diego, CA, USA) was used to prepare libraries that were sequenced by Shanghai Personal Biotechnology Co., Ltd. (Shanghai, China). The differentially expressed genes (DEGs) were identified by DESeq2 with an adjusted *p* value < 0.05 and absolute value of fold change (FC) > 1 or <−1. The Gene Ontology (GO) and Kyoto Encyclopedia of Genes and Genomes (KEGG) modules were used to determine the pathway enrichment of the DEGs, and *p* < 0.05 was defined as significantly enriched. A weighted correlation network analysis (WGCNA) was conducted to identify clusters (modules) of highly correlated genes. The modules were constructed with a cut height of 0.8, and the module eigengenes were subjected to interaction network analysis.

### 2.13. Statistical Analyses

Statistical analyses were performed to determine the significance of differences between groups by one-way or two-way analysis of variance (ANOVA) with GraphPad Prism software (version 6.0, San Diego, CA, USA). The data are shown as the mean ± SD. Significant differences were denoted by * *p* < 0.05, ** *p* < 0.01, *** *p* < 0.001, and **** *p* < 0.0001. ns = not significant.

## 3. Results

### 3.1. Expression of Recombinant Proteins

The Western blot results showed that the recombinant proteins chIFN-γ, chCD154, and chIFN-γ-chCD154 were displayed as a single band at 19, 16, and 35 kDa, respectively (Figure 1B). The results obtained in this study were consistent with the gel bands observed after SDS-PAGE (Figure 1A), which proved that the IPTG-induced expression of the recombinant proteins, chIFN-γ, chCD154, and chIFN-γ-chCD154, was successful. Approximately 1 mg amounts of pure recombinant protein of chIFN-γ, chCD154, and chIFN-γ-chCD154 were prepared.

### 3.2. ChIFN-γ-chCD154 Pretreatment Mitigated S. Typhimurium Infection in Chickens

The schematic illustration of the animal experimental design is shown in Figure 1C. Compared with the negative control, *S.* Typhimurium infection of chickens caused a significant loss of weight gain rate (Figure 1D) and lower survival rate (Figure 1E). ChIFN-γ, chCD154, or chIFN-γ-chCD154 pretreatment ameliorated the intestinal damage induced by *S.* Typhimurium infection, resulting in an increase in survival (Figure 1D) and weight gain rate (Figure 1E), especially in the chIFN-γ-chCD154 group. This suggests that chIFN-γ and chCD154 pretreatment exerted a synergistic protective effect on the *S.* Typhimurium-infected chickens.

In addition, the viability of *S.* Typhimurium can be assessed by its presence in fecal material and its translocation to the liver, spleen, and cecum [27]. *S.* Typhimurium infection induced high colonization levels of bacteria in the organs and large numbers of the bacteria in the feces of infected chickens. In contrast, compared with the non-pretreated group, chIFN-γ, chCD154, and chIFN-γ-chCD154 pretreatment decreased the load of *S.* Typhimurium in chickens, but the bacterial load of the chIFN-γ-chCD154 group showed the lowest level (Figure 1F).

### 3.3. ChIFN-γ-chCD154 Pretreatment Attenuated Inflammation in the S. Typhimurium-Infected Chickens

**Figure 2:**
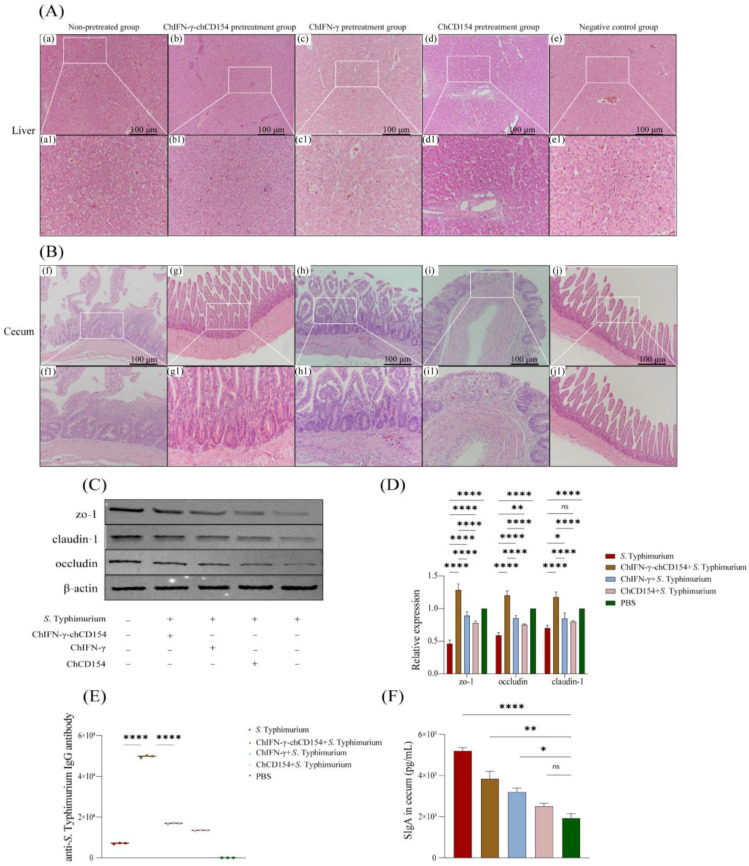
ChIFN-γ-chCD154 pretreatment attenuated *S.* Typhimurium-induced cecal inflammation. (**A**) Histological examination of the liver (bar = 100 μm). (**B**) Histological examination of the cecum (bar = 100 μm). (**C**) Protein expression of zo-1, claudin-1, and occludin. (**D**) The mRNA expression of zo-1, claudin-1, and occludin (n = 3 per group). (**E**) Levels of anti-*S.* Typhimurium IgG antibody in chickens. (**F**) The sIgA concentration in the cecum (n = 3 per group). The data are expressed as the mean ± SD (n = 3 per group), and a one-way ANOVA test was performed, followed by Tukey’s test. * *p* < 0.05, ** *p* < 0.01, **** *p <* 0.0001, ns = not significant.

The histopathological changes in the livers of infection-group chickens showed loosely arranged, vacuole-like structures, and inflammatory cell infiltration (Figure 2A). The ceca of the non-pretreated group chickens showed large numbers of infiltrating inflammatory cells, and the lamina propria were separated from the mucosal layer in localized areas (Figure 2B). However, chIFN-γ, chCD154, and chIFN-γ-chCD154 pretreatment reduced the tissue damage, especially in the chIFN-γ-chCD154 group.

The WB results (Figure 2C) revealed that in comparison with the negative control group, expression of the TJ proteins, zo-1, claudin-1, and occludin, was dramatically decreased in the non-pretreated chickens; however, chIFN-γ, chCD154, and chIFN-γ-chCD154 pretreatment significantly upregulated the expression of these three proteins in *S.* Typhimurium-infected chickens, especially the chIFN-γ-chCD154 group. The results of the RT-qPCR analysis of the expression of zo-1, occludin, and claudin-1 mRNA (Figure 2D) were consistent with the protein expression data.

### 3.4. ChIFN-γ-chCD154 Pretreatment Increased the Humoral Immune Response in S. Typhimurium-Infected Chickens

As shown in Figure 2E, anti-*S.* Typhimurium-specific IgG antibody levels were significantly elevated (*p* < 0.05) in the non-pretreated group compared with the negative control. Compared with the negative control group, chIFN-γ, chCD154, or chIFN-γ-chCD154 pretreatment remarkably increased anti-*S.* Typhimurium-specific IgG antibody levels (*p* < 0.05), especially in the chIFN-γ-chCD154 group. These findings indicated that chIFN-γ-chCD154 synergistically enhanced anti-*S.* Typhimurium-specific antibody levels in the infected chickens.

The non-pretreated group showed a higher sIgA level in comparison with the negative control group; the chIFN-γ-chCD154 group displayed the highest sIgA level (Figure 2F, *p* < 0.05). There were significant cooperative effects in the production of sIgA by the chIFN-γ-chCD154 group.

### 3.5. ChIFN-γ-chCD154 Pretreatment Reshaped the S. Typhimurium-Induced Intestinal Microbiota

**Figure 3:**
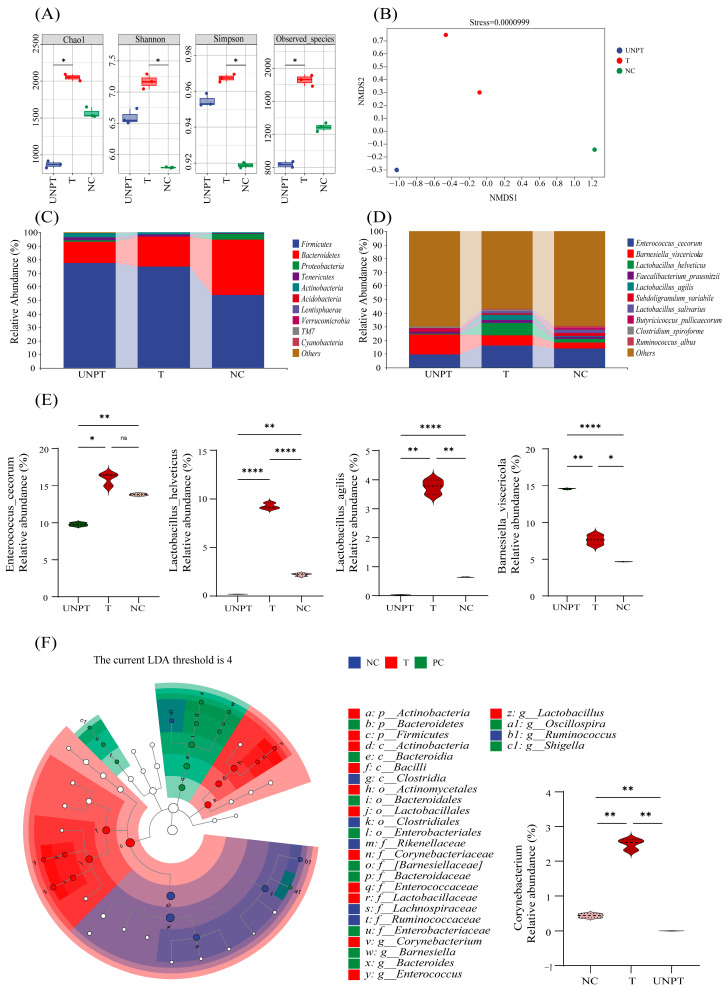
ChIFN-γ-chCD154 pretreatment alleviated intestinal damage in *S.* Typhimurium-infected chickens. (**A**) Alpha diversity indicators. (**B**) NMDS analysis of beta diversity in different groups (stress = 0.0000999). Relative abundance of bacterial phyla in cecal contents. (**C**) Relative abundance of bacterial genera in cecal contents (**D**). (**E**) Significance analysis of four different genera. (**F**) LEfSe analysis in different groups. The data are expressed as the mean ± SD (n = 3 per group), and a one-way ANOVA test was performed, followed by Tukey’s test. * *p* < 0.05, ** *p* < 0.01, **** *p* < 0.0001, ns = not significant.

The Chao1 index and the observed species parameter were significantly lower in the non-pretreated group relative to the negative control and the chIFN-γ-chCD154 groups (Figure 3A), while the Shannon and Simpson indexes were lower in the negative control group compared with those in the non-pretreated and chIFN-γ-chCD154 pretreatment groups (Figure 3A, *p* < 0.5). With regards to beta diversity, the NMDS analysis showed that the negative control, chIFN-γ-chCD154 pretreatment, and the non-pretreated groups were distinctly separated (stress = 0.0000999, Figure 3B). At the phylum level, Firmicutes, Bacteroidetes, Actinobacteria, and Proteobacteria were the dominant groups (Figure 3C). Compared with the negative control, *S.* Typhimurium infection dramatically reduced the relative abundance of Firmicutes and Bacteroidetes and increased the abundance of Proteobacteria (Figure 3C). However, chIFN-γ-chCD154 pretreatment significantly increased the relative abundance of Firmicutes and decreased the proportion of Proteobacteria relative to the non-pretreated group (Figure 3C). At the genus level, in comparison with the negative control group, the non-pretreated group displayed a significantly lower relative abundance of *Enterococcus_cecorum* (*p* < 0.0001), *Lactobacillus_helveticus* (*p* < 0.01), and *Lactobacillus_agilis* (*p* < 0.0001), while the relative abundance of *Barnesiella_viscericola* (*p* < 0.01) was significantly higher (Figure 3D,E). However, the relative abundance of these four genera returned to normal in the chIFN-γ-chCD154 group. Linear discriminant analysis effect size (LEfSe) with an LDA score > 4.0 was used to prove that the inflammation-inhibiting *Corynebacterium* was the indicator genus of the chIFN-γ-chCD154 group (Figure 3F, *p* < 0.01).

### 3.6. ChIFN-γ-chCD154 Pretreatment Altered the Transcriptome of Cecal Tissues in S. Typhimurium-Infected Chickens

**Figure 4:**
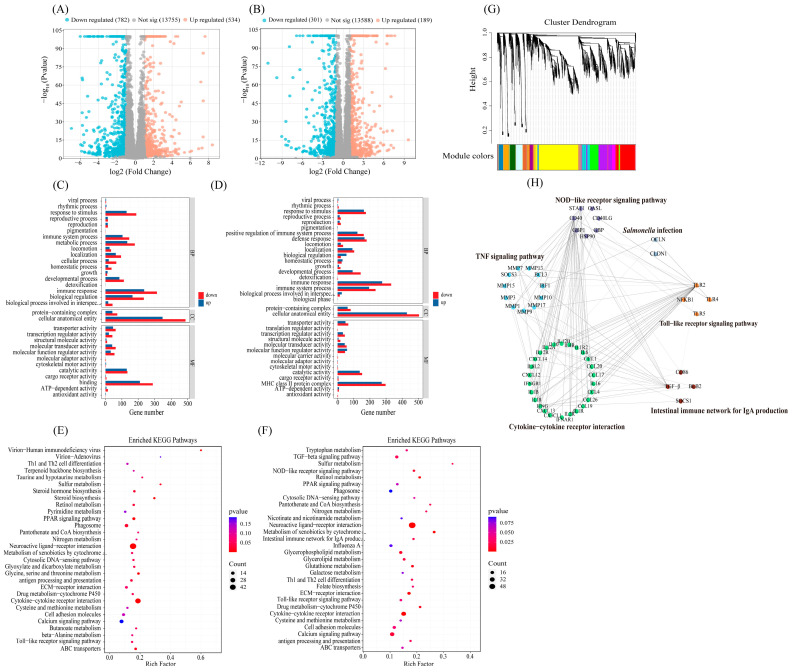
ChIFN-γ-chCD154 pretreatment altered the transcriptome expression profile in cecal tissues of *S.* Typhimurium-infected chickens. The volcano plot depicts the up-, down-, and nonregulated genes between the two groups, UNPT (non-pretreated) vs. NC (**A**) and UNPT vs. T (**B**). (**C**) Gene Ontology (GO) enrichment analysis of the DEGs, UNPT vs. NC, and (**D**) UNPT vs. T. (**E**) KEGG enrichment analysis of the DEGs, UNPT vs. NC, and (**F**) UNPT vs. T. (**G**) Gene clustering tree (dendrogram) based on hierarchical clustering of genes with adjacency-based dissimilarity. The distinctive color band designates the results obtained from the automatic single-block analysis. n = 6 per group. (**H**) The integrated interaction network comprises significantly enriched signaling pathways and the genes they contain. The node size represents the number of genes enriched in the pathway. n = 6 per group.

Compared with the negative control group, *S.* Typhimurium infection resulted in 1316 DEGs (534 upregulated and 782 downregulated) in the non-pretreated group (Figure 4A). In the chIFN-γ-chCD154 pretreatment group, 189 upregulated and 301 downregulated DEGs were identified in comparison with the non-pretreated group (Figure 4B). The GO analysis results showed that DEGs were significantly enriched in some biological processes. The abundant DEGs in the negative control and the non-pretreated groups were enriched in the regulation of immune responses and biological functions (Figure 4C), whereas DEGs in the chIFN-γ-chCD154 and the non-pretreated groups were mainly enriched in immune responses, immune system processes, defense responses, response to stimuli, and positive regulation of immune system processes (Figure 4D). The DEGs in the non-pretreated and the negative control groups displayed evident enrichment in the immune-related KEGG pathways, including cytokine–cytokine receptor interactions, Toll-like receptor signaling pathways, and Th1 and Th2 cell differentiation (Figure 4E). In the non-pretreated and the chIFN-γ-chCD154 pretreatment groups, the KEGG analysis suggested that DEGs were substantially enriched in immune-related pathways, including cytokine–cytokine receptor interaction, the Toll-like receptor signaling pathway, the NOD-like receptor signaling pathway, and the intestinal immune network for IgA production (Figure 4F).

A total of 25,489 genes (Table S1) for 18 samples were used to build a WGCNA. The WGCNA analysis identified 40 distinct modules with a mergeCutHeight of 0.8 (Figure 4G, Table S1). Six signaling pathways significantly related to the immune system were screened by the KEGG analysis (Table S1), including the NOD-like receptor signaling pathway, cytokine–cytokine receptor interaction, the intestinal immune network for IgA production, the Toll-like receptor signaling pathway, the TNF signaling pathway, and *Salmonella* infection. A network analysis identified an interaction between the Toll-like receptor pathway and *Salmonella* infection via TLR4 and TLR2, the NOD-like receptor pathway and *Salmonella* infection via GBP1, and the TNF signaling pathway and *Salmonella* infection via IRF1 (Figure 4H).

### 3.7. ChIFN-γ-chCD154 Pretreatment Suppressed the NF-κB Signaling Pathway to Reduce the Chicken Intestinal Inflammation Induced by S. Typhimurium

**Figure 5:**
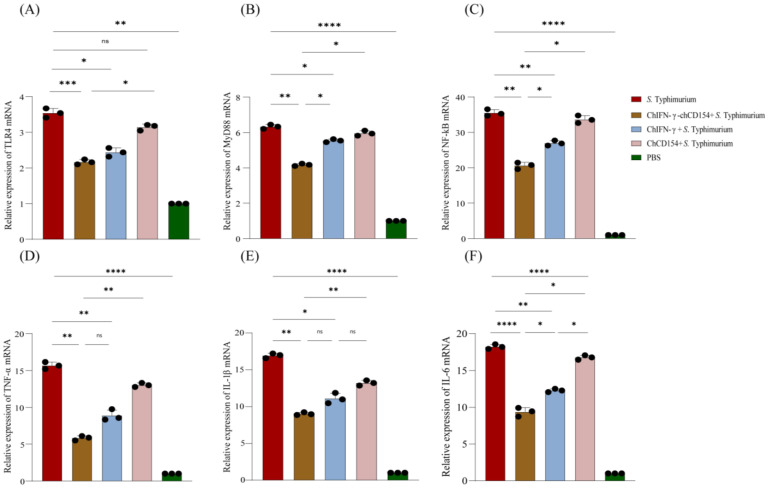
ChIFN-γ-chCD154 pretreatment suppressed the TLR4/Myd88/NF-kB pathway in the cecum of *S.* Typhimurium-infected chickens. The mRNA expression of (**A**) TLR4, (**B**) MyD88, (**C**) NF-kB, (**D**) TNF-α, (**E**) IL-1β, and (**F**) IL-6 in cecal tissues from *S.* Typhimurium-infected chickens. n = 3 per group. Data are expressed as the mean ± SD, and a one-way ANOVA test was performed, followed by Tukey’s test. * *p* < 0.05, ** *p* < 0.01, *** *p* < 0.001, **** *p* < 0.0001, ns = not significant.

In comparison with the negative control, *S.* Typhimurium infection significantly increased the mRNA expression of TLR4 (Figure 5A), MyD88 (Figure 5B), and NF-κB (Figure 5C) in the ceca of non-pretreated chickens (*p* < 0.01). In contrast, chIFN-γ, chCD154, or chIFN-γ-chCD154 pretreatment significantly decreased the mRNA expression of TLR4, MyD88, and NF-κB, and the chIFN-γ-chCD154 group showed the lowest level (*p* < 0.05). Similarly, the expressions of inflammation-related genes, including TNF-α (Figure 5D), IL-1β (Figure 5E), and IL-6 (Figure 5F), were significantly decreased in chIFN-γ, chCD154, or chIFN-γ-chCD154 pretreatment groups, especially the chIFN-γ-chCD154 group.

### 3.8. ChIFN-γ-chCD154 Pretreatment Promoted the IFN-Induced JAK/STAT1/IRF1/GBP1 Axis in the Cecum of Infected Chickens

**Figure 6:**
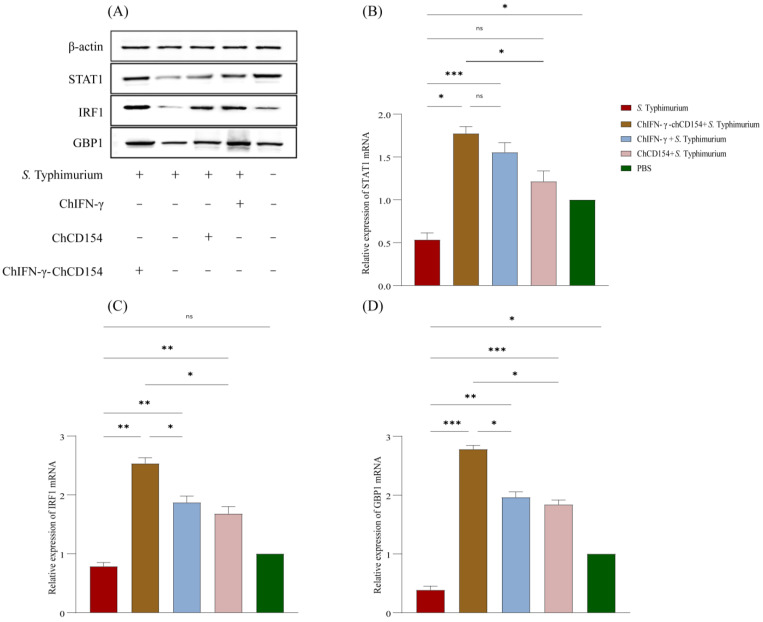
ChIFN-γ-chCD154 pretreatment promoted the IFN-induced JAK/STAT1/IRF1/GBP1 axis in the cecum of infected chickens. (**A**) Protein expression of STAT1, IRF-1, and GBP1. The mRNA expression of (**B**) STAT1, (**C**) IRF-1, and (**D**) GBP1 (n = 3 per group). Data are expressed as the mean ± SD, and a one-way ANOVA test was performed, followed by Tukey’s test. * *p* < 0.05, ** *p* < 0.01, *** *p* < 0.001, ns = not significant.

The WB protein analysis showed that compared with the negative control, the protein expression of STAT1, IRF1, and GBP1 decreased in the non-pretreated group (Figure 6A). Pretreatment with chCD154, chIFN-γ or chIFN-γ-chCD154 increased the expression of STAT1, IRF1, and GBP1 protein, especially the chIFN-γ-chCD154 group. Moreover, the mRNA expression trend of the above three genes (Figure 6B–D) in each group was consistent with the protein expression results.

## 4. Discussion

Here, we found that *S.* Typhimurium infection of chickens induced pathological changes to the intestinal epithelium, inflammation of the cecum, alteration of the cecal microbial community, and even death. Our findings also revealed that chIFN-γ-chCD154 pretreatment synergistically alleviated the inflammation, improved the survival rate of *S.* Typhimurium infected chickens, and significantly decreased the bacterial load in the feces and organs of the infected chickens. We conclude that pretreating chickens with the chIFN-γ-chCD154 fusion protein synergistically enhances the preventive effect of the individual proteins against *S.* Typhimurium infection.

*S.* Typhimurium infection disrupted the structure of the cecal epithelial mucosa, reducing its barrier effectiveness and inducing intestinal inflammation. The main tight junction proteins of the intestinal epithelial barrier, zo-1, occludin, and claudin-1, play an essential role in maintaining epithelial barrier integrity, and bacterial damage impairs the defense function of the intestinal epithelial cells [27,31]. The results of our study clearly demonstrated that *S.* Typhimurium infection considerably reduced the mRNA and protein expression of these TJ proteins. ChIFN-γ, chCD154, and chIFN-γ-chCD154 pretreatment significantly increased the expression of the TJ proteins, and the chIFN-γ-chCD154 group showed the highest level.

As a critical protective molecule, sIgA protects mucosal surfaces from the invasion and spread of pathogenic microorganisms and reduces intestinal inflammation caused by pathogen infection [32,33]. A significant rise of sIgA in the cecal tissue of the non-pretreated group was observed relative to the negative control group in response to the pathogen challenge. ChIFN-γ, chCD154, and chIFN-γ-chCD154 pretreatment increased the sIgA expression, with chIFN-γ-chCD154 providing the most protection.

The anti-*S.* Typhimurium IgG antibody level and the sIgA level showed a consistent trend in the different groups of this study, indicating that chIFN-γ-chCD154 pretreatment synergistically enhanced the humoral immune response and protected the intestinal mucosal barrier from damage. The intestinal structure plays a key role in the digestion and absorption of nutrients, and the invasion of pathogens caused intestinal villus damage and decreased absorption capacity, causing a reduction in the rate of weight gain. Compared with the individual proteins, the weight gain rate in the chIFN-γ-chCD154 group was higher. This could be attributed to the fact that chIFN-γ-chCD154 prevented the structural damage to the cecum caused by *S.* Typhimurium infection, thus alleviating its negative effects on digestion and nutrient absorption.

The results of transcriptome sequencing showed that chIFN-γ-chCD154 pretreatment downregulated TLR4 expression in cecal tissue compared with the non-pretreated group, which had a higher level of TLR4 compared with the negative control group. This finding was confirmed by RT-qPCR. LPS binding to TLR4 activates the downstream MyD88-dependent NF-κB inflammatory signaling pathway, increasing pro-inflammatory cytokine expression and inflammation [21]. The overproduction of TNF-α and IL-1β further decreases the expression of TJ proteins and increases the paracellular permeability of the intestinal barrier [21,34], which promotes the translocation of bacteria and the binding of LPS to TLR4. The activated TLR4/MyD88/NF-κB signaling pathway dramatically stimulates the excessive secretion of the pro-inflammatory cytokines, IL-1β, TNF-α, and IL-6, which further damages the intestinal epithelial barrier. Thus, the inhibition of TLR4, MyD88, and NF-κB expression and activation could effectively lower the release of inflammatory cytokines and dampen the host’s inflammatory response. Taken together, the results of our study support the hypothesis that chIFN-γ-chCD154 pretreatment significantly reduces the mRNA expression of TLR4, MyD88, and NF-κB in the cecum of *S.* Typhimurium-infected chickens. This inhibition downregulates the mRNA expression levels of TNF-α, IL-6, IL-12, and IL-1β. Pretreatment with chIFN-γ-chCD154 significantly upregulated the expression of TJ proteins in the cecal tissues of infected chickens, decreased secretion of pro-inflammatory cytokines, synergistically reduced intestinal inflammation, and alleviated the severe damage to the cecum caused by *S.* Typhimurium. In turn, the strengthening of the intestinal epithelial barrier inhibited activation of the TLR4/MyD88/NF-κB signaling pathway. Our study demonstrated that chIFN-γ-chCD154 pretreatment could inhibit the TLR4/Myd88/NF-κB signaling pathway and alleviate the *S.* Typhimurium-induced inflammation of the cecum.

Interferon regulatory factor 1 (IRF1) of chickens have been identified as key elements in managing IFN-γ’s involvement in the immune response. The CD154–CD40 interaction can upregulate the production of IFN-γ [35], and Moschonas et al. showed that CD154 could promote the expression of IRF1 [36]. Previous studies have shown that IFN-γ, via the JAK/STAT1 axis, induced the production of IRF1 and upregulated guanylate binding proteins (GBPs) to promote the disruption of *Salmonella*-containing vacuoles (SCVs) and the release of *Salmonella* into the cytoplasm, which triggered cell death, thus inhibiting proliferation of the pathogen [25,37]. GBPs are major players in host immunity and provide defense against infection [38]. In this study, chIFN-γ-CD154 pretreatment increased the survival of *S.* Typhimurium-infected chickens by inducing STAT1 and IRF1 and upregulating the production of GBP1, which decreased the bacterial load in organs and accelerated the clearance of *S.* Typhimurium. In the chIFN-γ-CD154 pretreatment group, the upregulation of IRF1 and GBP1 expression enhanced the innate immune defense system. Transcriptome sequencing revealed significant enrichment in the cytokine receptor interaction pathway, the TNF signaling pathway, and the NOD-like receptor signaling pathway between the chIFN-γ-CD154 and the non-pretreated groups, emphasizing the close involvement of chIFN-γ-CD154 in the immune response.

The intestinal microbiota are critical participants in regulating the host immune system, maintaining cecal epithelial integrity, and inhibiting inflammation in the gut, which is associated with alteration of the intestinal microbial community [39]. Infections with pathogens such as *Clostridium perfringens*, *Eimeria* species, and *S.* Typhimurium cause significant changes in the cecal microbiota [39]. Here, we showed that pretreatment with chIFN-γ-CD154 restored the cecal microbial community in *S.* Typhimurium-infected chickens by increasing the relative abundances of *Enterococcus_cecorum*, *Lactobacillus_helveticus*, and *Lactobacillus_agilis*, accompanied by a decrease in the relative abundances of *Barnesiella_viscericola*. As demonstrated by previous research, *Enterococcus_cecorum* is a normal inhabitant of the intestine of birds and other vertebrates [40], and the majority of *Lactobacillus* species have been confirmed to alleviate oxidative stress, inhibit production of inflammatory cytokines, and help to restore the host’s damaged mucosal epithelial barrier [41]. *Lactobacillus helveticus* was shown to modulate the host immune response, prevent gastrointestinal infections, enhance protection against pathogens, and positively affect the composition of the intestinal microbiota [42]. These activities restored the balance between innate and adaptive immunity, repaired damaged immune system organs, strengthened the mucosal barrier, and augmented antimicrobial immune functions [42]. Baldwin et al. showed that *Lactobacillus_agilis* could improve weight gain, modify the intestinal microbiota, and decrease the numbers pathogenic taxa in chickens [43]. These observations are consistent with our study. *Barnesiella viscericola* is associated with beneficial effects, such as protection against damage from intestinal inflammation [44]. The relative abundance of *Barnesiella viscericola* in *S.* Typhimurium-infected chickens treated with the chIFN-γ-CD154 fusion protein was restored to levels similar to those observed in the negative control chickens. The results of this study demonstrated that chIFN-γ-CD154 is involved in the maintenance of beneficial gut bacteria that preserve the integrity of the chicken intestinal barrier, which is in line with the previous research [45].

In summary, the current study revealed that *S.* Typhimurium infection reduced the relative abundance of beneficial bacteria, disrupted the cecal epithelial barrier, and caused excessive inflammation in chickens. Pretreatment with chIFN-γ-chCD154 ameliorated these symptoms through critical interactions with the TLR4/MyD88/NF-κB signaling pathway and the IFN-γ/STAT1/IRF1/GBP1 axis. Our findings provide novel insights into the regulatory mechanism of chIFN-γ-chCD154 and may contribute to the deployment of more effective antimicrobial therapies and preventive strategies to inhibit bacterial infection.

## 5. Conclusions

In conclusion, this study found that chIFN-γ-chCD154 can provide effective protection against *S.* Typhimurium infection in chickens, and its mechanism of action paves the way for exploring new strategies to prevent infection by specific bacterial pathogens.

## Figures and Tables

**Table 1:** Paired primers for generation of the recombinant plasmids.

Name	Primer (5′-3′)
IFN-γ F	5′-GAGAGCTCGGTACCCTCGAGATGCATACTGCAAGTAGTCT-3′
IFN-γ R	5′-ATCTAGACTGCAGGTCGACAAGCTTAGAACCACCGGATCCGAGATTGTCGACGCAATTGC-3′
CD154 F	5′-GAGAGCTCGGTACCCTCGAGATGAATGAAGCCTACAGCCC-3′
CD154 R	5′-TATCTAGACTGCAGGTCGACAGAACCACCGGATCCCTACAGCTTGAACATGCCAA-3′
IFN-CD154 F	5′-ATCTCGGATCCGGTGGTTCTATGAATGAAGCCTACAGCCC-3′
IFN-CD154 R	5′-TTTTAAGCAGAGATTACCTACTACAGCTTGAACATGCCAA-3′

**Table 2:** Paired primers for RT-qPCR of genes.

Gene	Forward Primer (5′-3′)	Reverse Primer (5′-3′)	Reference
TLR4	TGCCATCCCAACCAACCACACACAG	ACACCCACTGAGCAGCACCAA	[27]
MyD88	AGAAGGTGTCGGAGGATGGTG	GGGCTCCAAATGCTGACTGC	
IL-1β	GGTCAACATCGCCACCTACA	CATACGAGATGGAAACCAGCAA	
TNF-α	AGATGGGAAGGGAATGAACC	TCAGACATCAAACGCAAAAG	
IL-6	CAAGGTGACGGAGGAGGAC	TGGCGAGGAGGGATTTCT	[28]
zo-1	TAAAGCCATTCCTGTAAGCC	AAGCATCCTCTTCAAAGTCTG	
Claudin-1	CTGATTGCTTCCAACCAG	ATTGATGGTGGCTGTAAAGAG	
Occludin	TCATCGCCTCCATCGTCTAC	GCACAAAGATCTCCCAGGTC	
β-actin	CCACCGCAAATGCTTCTAAAC	AAGACTGCTGCTGACACCTTC	
NF-κB	ACCCCTTCAATGTGCCAATG	TCAGCCCAGAAACGAACCTC	
STAT1	CGTATCTTTTGCTACAGTGCT	TTTGCTTTTCCTTATGTTGTG	[29]
IRF1	CCTGACATTGAAGAAGTGAAG	TCTGCTGACTCCTCCATC	
GBP1	AAGTCCTTCCTGATGAACC	CTTGGTCTCCGCATACAC	[30]

## Data Availability

The raw sequencing reads were deposited into the database URL: https://www.ncbi.nlm.nih.gov/sra/?term=PRJNA944214 (accessed on 17 September 2024). The other original contributions presented in this study are included in the article/Supplementary Materials. Further inquiries can be directed to the corresponding authors.

## Supplementary Materials

The following supporting information can be downloaded at: https://www.mdpi.com/article/10.3390/vetsci12020112/s1, Table S1: Transcriptome data statistics of chicken cecum and the original images of Western Blots.
