# Supplementary material for: Administration of a Recombinant Fusion Protein of IFN-γ and CD154 Inhibited the Infection of Chicks with Salmonella enterica
Source: Vet Sci. 2025 Feb 2;12(2):112. doi: 10.3390/vetsci12020112 (PMC11861687; doi:10.3390/vetsci12020112)
Supplement: Supplementary file 1 [file vetsci-12-00112-s001.zip › vetsci-3299240-original-images.pdf]

The origin uncropped blots of **Figure 1**.

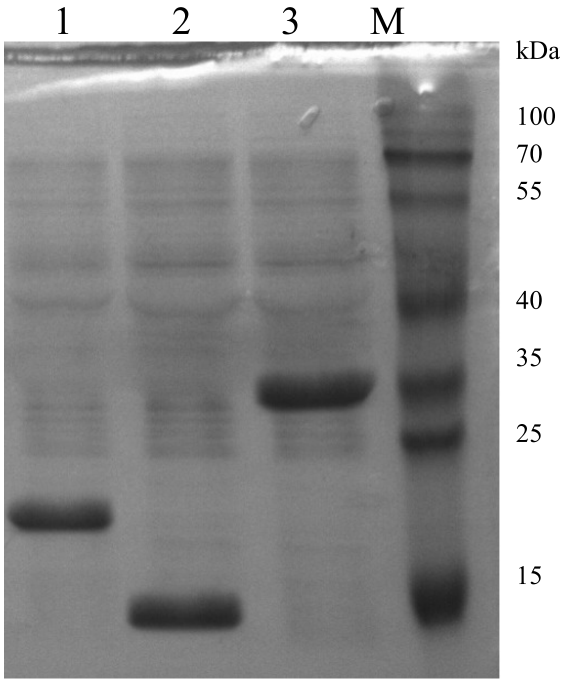

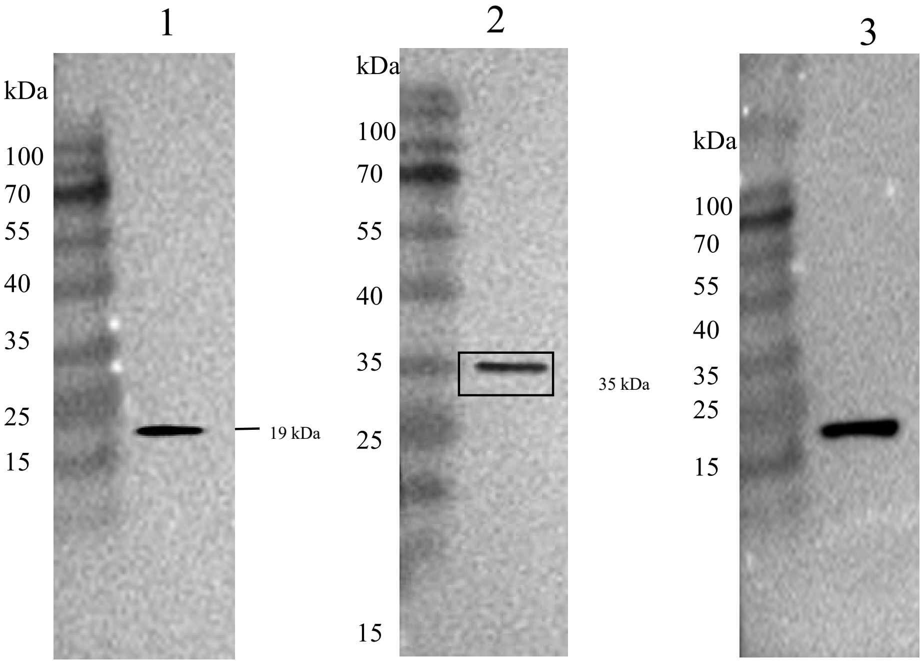

Lane 1: chIFN-γ
Lane 2: chIFN-γ-chCD154
Lane 3: chCD154

The origin uncropped blots of **Figure 2**.

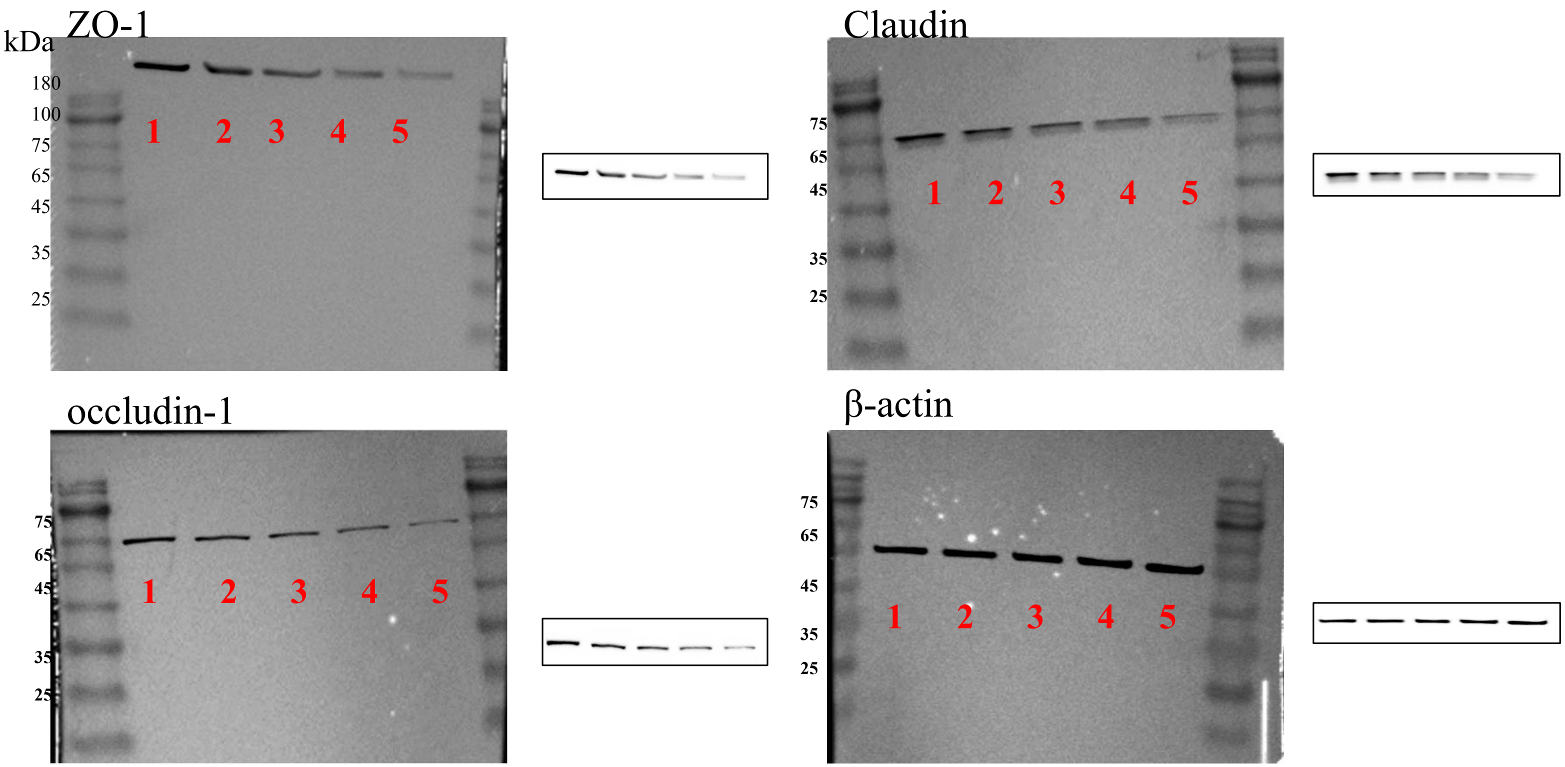

Lane 1: the negative control group
Lane 2: chIFN-γ-chCD154 pretreatment group
Lane 3: chIFN-γ pretreatment group
Lane 4: chCD154 pretreatment group
Lane 5: the non-treated group

The origin uncropped blots of **Figure 6**.

β-actin

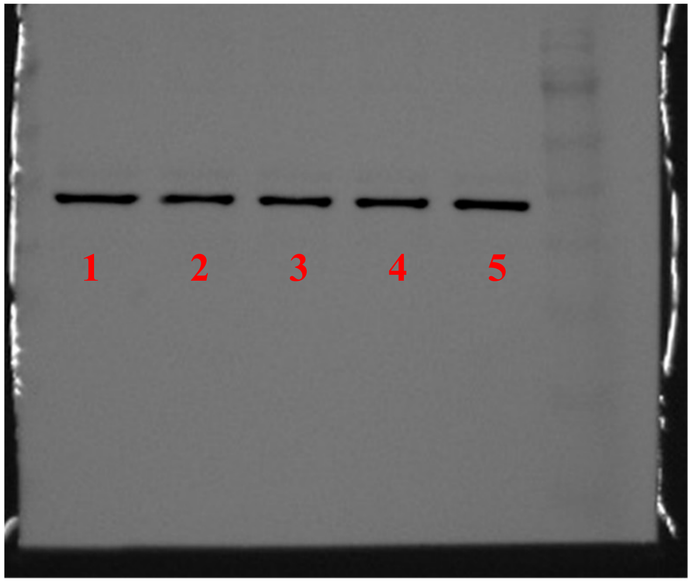

STAT1

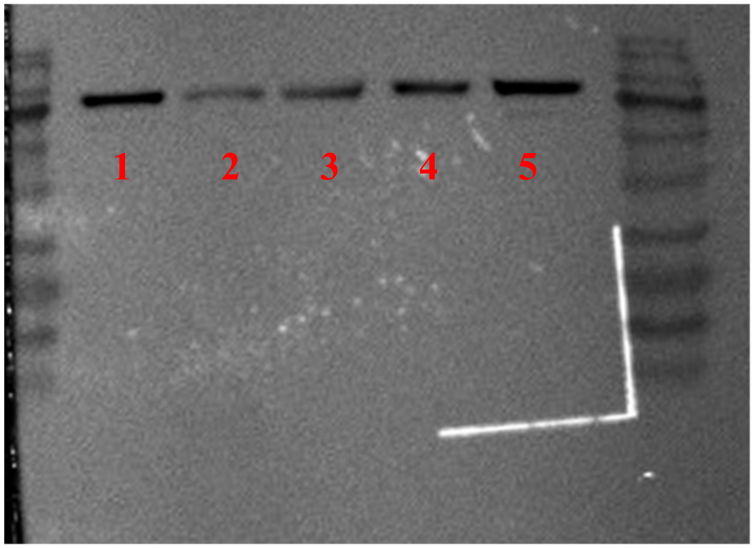

Lane 1: chIFN-γ-chCD154 pretreatment group
Lane 2: the non-treated group
Lane 3: chCD154 pretreatment group
Lane 4: chIFN-γ pretreatment group
Lane 5: the negative control group

The origin uncropped blots of **Figure 6**.

GBP 1

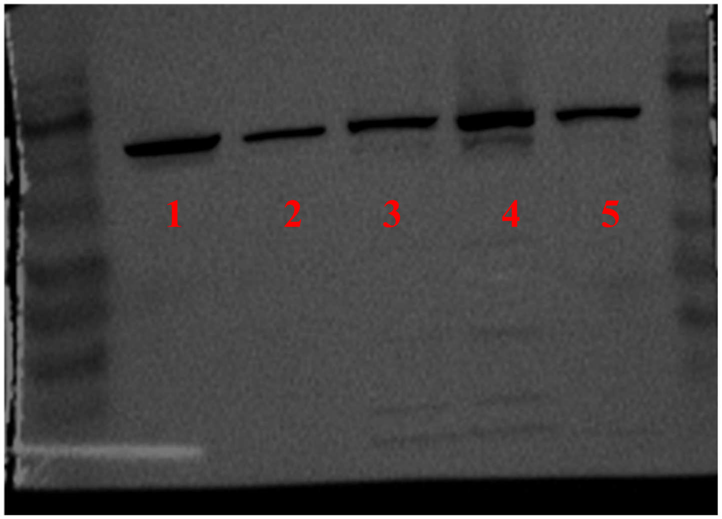

IRF1

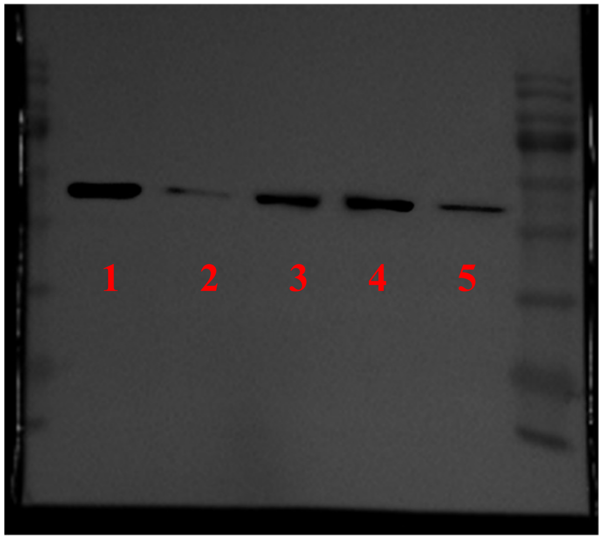

Lane 1: chIFN-γ-chCD154 pretreatment group
Lane 2: the non-treated group
Lane 3: chCD154 pretreatment group
Lane 4: chIFN-γ pretreatment group
Lane 5: the negative control group
